# Evaluation of Valorisation Strategies to Improve Spent Coffee Grounds’ Nutritional Value as an Ingredient for Ruminants’ Diets

**DOI:** 10.3390/ani13091477

**Published:** 2023-04-26

**Authors:** David San Martin, Jone Ibarruri, Nagore Luengo, Jorge Ferrer, Aser García-Rodríguez, Idoia Goiri, Raquel Atxaerandio, Mounir Medjadbi, Jaime Zufía, Estíbaliz Sáez de Cámara, Bruno Iñarra

**Affiliations:** 1 AZTI, Food Research, Basque Research and Technology Alliance (BRTA), Astondo Bidea, Edificio 609, 48160 Derio, Spain; jibarruri@azti.es (J.I.); nluengo@azti.es (N.L.);; 2 NEIKER, Basque Institute for Agricultural Research and Development, Basque Research and Technology Alliance (BRTA), Campus Agroalimentario de Arkaute s/n, 01192 Arkaute, Spain; 3 Faculty of Engineering Bilbao, University of the Basque Country (UPV/EHU), Ingeniero Torres Quevedo Plaza, 1, 48013 Bilbao, Spain

**Keywords:** food waste, grinding, enzymatic hydrolysis, animal feed, circular economy, upcycling

## Abstract

**Simple Summary:**

More than 10 million tons of coffee are consumed annually in the world, generating two kg of wet spent coffee grounds per kg of coffee consumed, which are considered food waste. Despite the interesting nutritional value of spent coffee grounds for ruminant feeds, their fibre fraction is very high, which presents a limitation for including this alternative ingredient in animals’ diets due to its low digestibility. This study considered thermal and mechanical treatments combined with enzymatic hydrolysis to improve the spent coffee grounds’ nutritive value and digestibility. The main conclusions are that the effect of enzymatic treatments is overwhelmed by the action of ruminal bacteria and that diminution of the particle size is the best strategy to improve the spent coffee grounds’ digestibility.

**Abstract:**

Lignin in animal diets is a limiting factor due to its low digestibility. This study assessed the effects of thermal or mechanical pre-treatments and enzymatic hydrolysis on spent coffee grounds’ (SCG) nutritional value and digestibility. A first trial studied the effect of thermal pre-treatment and hydrolysis with removal of the liquid part and a second trial studied mechanical pre-treatment and hydrolysis with and without removal of the liquid part. Autoclaving did not improve the enzymatic performance nor the nutritional value. Hydrolysis reduced the digestibility of the solid phase and impaired its ruminal fermentation efficiency. Hydrolysates without removing the liquid part improved its nutritional value, but not compared with unprocessed SCG. Grinding increased crude protein and reduced crude fibre and protein, which led to greater fermentation and in vitro digestibility. Thus, grinding emerges as the most promising valorisation strategy to improve SCG nutritional characteristics and their use for animal feed, contributing to the circular economy.

## 1. Introduction

Coffee is one of the world’s most important commodities and its consumption is widespread across the planet. According to the International Coffee Organization [1], about 10.2 million tons of coffee were consumed worldwide during the 2020/2021 period (coffee year: October–September). This amount represents an increase of 3.27% compared with 2019/2020, despite the influence of the COVID crisis on food consumption. Europe is the region with the highest consumption worldwide, at 3.3 million tons of coffee.

The hotels, restaurants, and catering industry (HORECA) is one of the most important sectors responsible for this consumption since its activity consists basically of preparing and serving food and beverages. However, coffee consumption involves the production of different organic wastes. The most important of these are spent coffee grounds (SCG), which are the insoluble parts that remain after making coffee. Each kg of coffee consumed produces two kg of wet SCG that are considered food waste (about 6.6 million tons of SCG) and should be managed in the best possible way [2].

The most extensively used method of managing SCG in Europe is landfilling [3], which is limited by a current European directive (Directive EU 2018/850). In any case, the management of SCG in landfill involves a carbon footprint of about 1716 million kg CO_2_eq/year, associated with an environmental cost of 0.26 kg CO_2_eq/kg of food waste [4]. Therefore, it is necessary to find a global solution for the reintroduction of this by-product into the value chain, avoiding the environmental impact of its management as waste.

Several potential alternatives have been considered for the recycling of SCG, such as the production of pellets as an energy source or as a substrate for biodiesel production [5,6]. However, these alternatives could be considered of low value according to the prioritisation hierarchy for the best use of surplus food, as established by the EU Waste Framework Directive 2008/98/EC: first reduce food surplus, followed by its use for human consumption or livestock feed, and finally for bioenergy such as biogas. Furthermore, if the nutritional properties of this raw material are considered, they are suitable for higher value applications such as animal feed. The high content of cellulose, hemicelluloses, proteins, fats, polyphenols, and minerals makes SCG interesting for the livestock sector [7,8].

A previous study proposed the use of the existing logistic routes for HORECA waste, such as those for used oil, to collect the SCG. This framework established a collection period of no more than 4 days depending on microbiological stability. In addition, it stipulates periodic cleaning of the containers and their placement in areas not intended for garbage [2].

According to the European Feed Manufacturers’ Federation (FEFAC), farm animals in Europe consumed an estimated 701 million tons of feed in 2021 [9], 22% produced by compound feed manufacturers. In 2020, compound feed production in the European Union reached 150.2 million tons of feed, excluding petfood [10].

The main cost factor in livestock activity is animal feeding, with up to 55% for poultry, 32% for pigs, and 14% for cattle [10]. In addition, feed costs have increased more than production prices in recent years. Therefore, farmers need to improve their productivity to maintain the sustainability and profitability of livestock activity in the future.

Within this framework, despite the interesting nutritional value of SCG, its fibre fraction is very high and its acid detergent lignin (ADL) content is around 27.83% [2]. This ADL content presents a limitation for including this alternative ingredient in animals’ diets due to its low digestibility, as reported in previous studies [2,11,12]. Therefore, it is necessary to degrade lignocellulosic bounds to allow a higher level of inclusion of SCG ingredients in animal feed.

In this context, thermal and mechanical pre-treatments are presented as effective strategies to break the lignocellulosic bounds in the fibre fraction, while increasing the surface area of the material and facilitating contact with enzymes [13]. This would lead to an increase in the digestibility of the ingredients [14]. However, the intensity and duration of thermal treatments have an important effect on the final digestibility [15]. Mechanical pre-treatment aims to reduce the particle size of substrates, which normally leads to more digestible ingredients [16,17,18]. However, defining the optimal particle size of SCG is of utmost importance to achieve its optimal nutrient and energy digestibility as an ingredient.

Meanwhile, enzymatic hydrolysis has the potential to increase digestibility by degrading fibre fractions [19]. Hydrolysis involves the breaking of bonds to obtain fibres of different sizes. This process must always be adapted to the characteristics of both the initial product to be hydrolysed and the final product to be obtained. Thus, the optimum conditions of the hydrolysis treatment are critical and must be defined.

The objective of the present study was to determine the best process to improve the digestibility of SCG, with the aim of increasing their inclusion in ruminants’ diets. Thus, this study focused on evaluating the effects of two different pre-treatments (autoclaving and grinding) and enzymatic hydrolysis using different enzymes on the nutritive value, in vitro organic matter digestibility (IVOMD), and short chain fatty acids (SCFA) production of SCG from the HORECA sector.

## 2. Materials and Methods

### 2.1. Experimental Designs

The SCG samples used in this study were obtained from HORECA industry in northern Spain (Basque Country). The initial samples were divided into 500 g bags and were kept frozen (−20 °C) until processing. Two different experiments were designed and performed: the first was to evaluate the effects of thermal pre-treatment of the SCG and hydrolysis with four different enzymes removing the liquid phase, whereas the second studied the effects of mechanical pre-treatment and hydrolysis with a mix of two enzymes selected accordingly to the results of the first experiment with and without removing the liquid phase.

#### 2.1.1. Thermal Pre-Treatment and Enzymatic Hydrolysis

In the first experiment, different cellulolytic enzymes were used to degrade the SCG fibre fractions. Thermal pre-treatment was also evaluated as a method to facilitate the availability of cellulose and hemicellulose fractions to the enzymes.

The trial was designed as a factorial design (2 × 5) including two factors: thermal pre-treatment (with and without) and enzymatic hydrolysis (EH) (CTR: unprocessed SCG; 1: Celuclast^®^; 2: Ultimase^®^; 3: Viscozyme^®^; 4: Ultraflo^®^).

The initial SCG sample was divided into two. Half of the sample was preserved for further analysis. The other half was subjected to thermal pre-treatment (autoclaving) at 121 °C for 15 min. Both subsamples were again divided into two halves. One half of each subsample was preserved unmodified for further analysis. The other half was divided into four subsamples which were hydrolysed by four different enzymes.

Enzymes were provided by Novozymes (NovozymesA/S, Bagsvaerd, Denmark). Viscozyme^®^ is an endo-beta-glucanase that hydrolyses (1,3)- or (1,4)-linkages in beta-D-glucans with high mannase activity. Celluclast^®^ and Ultimase^®^ are cellulases that hydrolyse (1,4)-beta-D-glucosidic linkages in cellulose and other beta-D-glucans. Ultraflo^®^ is an endo-beta-glucanase that hydrolyses (1,3)- or (1,4)-linkages in beta-D-glucans and a xylanase that hydrolyses (1,4)-beta-D-xylosidic linkages in xylans.

Hydrolysis conditions were stablished based on the technical data sheets for the enzymes provided by Novozymes: pH 5, 55 °C, 20 h, 250 rpm, ratio 1:1 SCG:water, and 1% (*v*:*w*) of enzyme with respect to fibre. Hydrolysis was performed using Sell Symphony 7100 Bathless Dissolution Distek equipment (Distek Inc., North Brunswick, NJ, USA), controlling and monitoring temperature, time, and stir speed. The pH of each run of the experiments was controlled manually and adjusted with NaOH 1 M in a final volume of 500 mL. The hydrolysis processes were ended by enzyme inactivation at a temperature of 90 °C for 15 min. Then, the samples were centrifuged (2650× *g*; 15 min; room temperature), and two fractions were recovered: the solid sample (the one intended for animal feed) and the liquid fraction (not considered for animal feed in this study; this fraction was obtained for all treatments except for the CTR which was unprocessed). This procedure was performed three separate times.

After all the treatments, samples were freeze-dried and kept in closed plastic bags until physicochemical analyses and IVOMD determination.

#### 2.1.2. Mechanical Pre-Treatment and Enzymatic Hydrolysis with and without Removing the Liquid Phase

In the second experiment, considering the results obtained in the first experiment, two of the enzymes used in the first trial were selected to perform the hydrolysis of the SCG (Viscozyme^®^ and Ultimase^®^). Additional evaluation assessed particle size reduction by means of grinding as pre-treatment to facilitate the availability of cellulose and hemicellulose fractions to the enzymes.

In addition, with the aim of testing the effect on IVOMD of the potential release of soluble components of SCG to the liquid fraction in the hydrolysis process, a hydrolysate sample without separation of the solid and liquid fraction was also evaluated in this experiment. Thus, the hydrolysis treatments evaluated in this experiment were: unprocessed SCG, SCG hydrolysed with the mixture of the two described enzymes and with the liquid fraction removed, and SCG hydrolysed with the mixture of the two described enzymes without removing the liquid fraction.

The trial was designed as a factorial design (2 × 3), with two factors: grinding (unground sample; coarse grinding and fine grinding) and hydrolysis (without hydrolysis, hydrolysis without removing the liquid fraction, and hydrolysis with removal of the liquid fraction).

The initial SCG sample was divided into three. One of the samples was preserved for further analysis. The other two were subjected to grinding in a Comitrol^®^ Processor Model 1700 (Urschel, Chesterton, IN, USA). One subsample was ground using a knife head with 160 blades to achieve a final estimated particle size of 250 µm, and the other subsample was ground using a knife head with 260 blades to achieve an estimated final particle size of 100 µm.

Each subsample was again divided into three. One of the samples was preserved unmodified for further analysis. The other two were hydrolysed by the mixture of two different enzymes (Viscozyme^®^ and Ultimase^®^, Novozymes A/S, Bagsvaerd, Denmark). 

Hydrolysis conditions were established based on the technical data sheets for the enzymes provided by Novozymes: pH 5, 55 °C, 20 h, 250 rpm, ratio 1:1 SCG:water, and 1% (*v*:*w*) of each enzyme with respect to fibre. The hydrolysis process, inactivation and centrifugation were performed as explained in Section 2.1.1. Then, all the treated samples were freeze-dried and kept in closed plastic bags until physicochemical analyses and IVOMD determination.

### 2.2. Rumen In Vitro Digestibility Determination

Each of the samples obtained in the first and second experiments were used as a substrate in a short-term in vitro batch fermentation trial as described by Pell and Schofield [20], to test the effects of the different treatments on the rumen IVOMD of the SCG and the fermentation characteristics.

Each of the samples were incubated in triplicate, in four different incubation runs performed in different weeks.

In each of the runs, rumen fluid was collected from one multiparous Latxa ewe slaughtered for production purposes. Before slaughtering, ewes were fed fescue hay ad libitum for 3 weeks and had free access to fresh water. Ruminal fluid was collected before the morning feeding and strained through four layers of cheesecloth into a pre-warmed thermos flask.

Approximately 500 mg of solid samples from the three independent processing runs were weighed into 125 mL serum bottles, 50 mL of culture fluid was added (1:4 ruminal fluid and phosphate–bicarbonate buffer, respectively) [21], and bottles were crimp sealed. Bottles were incubated at a constant temperature (39 °C) in an incubator for 24 h. Gas production was released at 2, 4, 6, 8, 10, 12, and 15 h post-inoculation to avoid pressure exceeding 48 kPa in the bottle headspace, as suggested by Theodorou et al. [22]. After 24 h of incubation, bottles were put into the fridge for 15 min to stop fermentation before subsequent sampling for SCFA determination.

IVOMD was calculated as described by Pell and Schofield [20]. In this process, 45 mL of a neutral detergent solution was added to each bottle and warmed at 105 °C for 1 h; then, the bottles were cooled, filtered through glass filter crucibles (Porosity 2) and washed with distilled water, ethanol, and acetone. The remaining sample was dried at 100 °C overnight and then burned in a muffle furnace at 525 °C and weighed to obtain true IVOMD values according to the difference from the weight of the incubated organic matter (OM) [20].

### 2.3. Chemical Analyses

The physicochemical composition was analysed only in the solid fractions of both experiments. SCG was analysed for dry matter (DM, method 934.01), ash (method 942.05), and nitrogen (method 984.13) content following the Association of Official Analytical Chemists [23]. Neutral detergent fibre (NDF) was determined by the UNE EN ISO 16472 method with use of an alpha amylase but without sodium sulphite, and was expressed free of ash. Acid detergent fibre (ADF) and ADL, expressed exclusive of residual ash, were determined by AOAC method 973.18. Neutral detergent insoluble protein (NDICP) and acid detergent insoluble protein (ADICP) were determined by analysing the NDF and ADF residues, respectively, for Kjeldahl nitrogen. Total reducing sugars (TRS) were determined by the dinitrosalicylic acid reagent method [24] adjusted for the microplate (Thermo Fisher Scientific, Roskilde, Denmark) assay procedure [25].

The antioxidant activity of samples was measured using the DPPH (2,2-Diphenyl-1-picrylhydrazyl, D9132 Sigma Aldrich, Steinheim, Germany) radical scavenging activity method [26]. DPPH in methanol (40 ppm) was prepared and 280 μL of this solution was added to 20 μL of sample solution. The mixture was incubated at room temperature in the dark for 30 min. Absorbance was measured at 515 nm. The standard comprised of water–methanol (50% *v*/*v*) and different concentrations of trolox (218940050, Acros Organics, NJ, USA). The antioxidant capacity was expressed as mg trolox equivalent antioxidant capacity (TEAC) per g of DM, using the calibration curve. TPC was measured using the Folin–Ciocalteu method [27]. Initially, 30 μL of Folin–Ciocalteu (J/4100/08, Fischer Scientific, Loughborough, UK) solution was added to 140 μL of sample, blank, or standard and 140 μL of Na_2_CO_3_ 7% (*w*/*v*) (Sigma Aldrich, Steinheim, Germany). The mixture was incubated at room temperature in the dark for 1 h and the absorbance was measured at 750 nm. Gallic acid (G7384, Sigma Aldrich, Steinheim, Germany) was used as standard at a concentration range of 1.4–20 ppm, and results were expressed as mg gallic acid equivalent (GAE) per g of DM sample.

For caffeine analysis, 1 g of previously homogenized sample was weighed into an Erlenmeyer flask and 2 g of magnesium oxide was added. The samples were diluted with milli Q water without exceeding a volume of 100 mL and boiled for 15 min. The samples were then made up to the volume of 100 mL, filtered through 0.45 m PTFE filters and placed in vials for subsequent caffeine analysis. Caffeine was measured using chromatographic assays on an Agilent Technologies 1200 series HPLC system (Santa Clara, CA, USA) equipped with a UV/Vis photodiode array detector, a quaternary pump, and a degasser system. The column used was a Nova Pak C—18 3.9 × 150 mm (Waters, Etten-Leur, The Netherlands) at 40 °C. The mobile phases used were acetonitrile 12% and water 88% in isocratic elution, and were pumped at 1.0 mL/min. In this process, 20 µL of each sample and standard were injected and absorbance was measured at 274 nm of wavelength. The standard used was caffeine and the constructed calibration curve ranged from 10 to 70 ppm. 

In the first experiment, TRS, caffeine, TEAC, and TPC were analysed only in the liquid fraction in order to quantify the release of compounds to the liquid fraction. In the second experiment, they were analysed only in the solid fraction in order to quantify their concentrations in the final ingredient intended for animal feed.

The analysis of the SCFA (acetic, propionic, butyric, isobutyric, valeric, and isovaleric) was performed by gas chromatography using a flame ionization detector as described by Goiri et al. [28]. Briefly, a volume of 4 mL of ruminal incubation medium mixed with 1 mL of a solution of 20 g/L of metyl-valeric acid as an internal standard in 0.5 N HCl was centrifuged (462× *g* for 20 min at 4 °C) and microfiltered (premium syringe filter regenerated cellulose, 0.45 µm 4 mm, Agilent Technologies, Madrid, Spain), and 0.5 µL of liquid phase was directly injected into the apparatus (Agilent 6890 N, Agilent, Spain). Data are expressed in mmol/100 mmol.

### 2.4. Calculations & Statistical Analysis

Data distribution was tested for normality using the Shapiro–Wilk test, and the Levene test was used to assess the equality of variances.

The data of the physicochemical characterization of the SCG solid fractions and the TRS, TPC, caffeine, and TEAC of the liquid fraction were processed by analysis of variance using the GLM procedure of SAS [29], including the fixed effects of the pre-treatment, hydrolysis, and the interaction between them.

In the first experiment, the total number of observations for the IVOMD trial comprised 3 runs of processing (hydrolysis) × 2 pre-treatment conditions × 5 enzymatic procedures × 4 in vitro incubation runs × 3 laboratory replicates = 360; however, after averaging the incubation runs and laboratory replicates, the remaining 30 observations were subjected to analysis of variance using the GLM procedure [29]. The statistical model included the fixed effects of the thermal pre-treatment, the enzymatic hydrolysis, and their interaction.

In the second experiment, the total number of observations for the IVOMD trial comprised 3 runs of processing (hydrolysis) × 3 grinding treatments × 3 hydrolysis procedures × 4 in vitro incubation runs × 3 laboratory replicates = 324; however, after averaging the incubation runs and laboratory replicates, the remaining 27 observations were subjected to analysis of variance using the GLM procedure [29]. The statistical model included the fixed effects of grinding, hydrolysis, and their interaction.

Least squares mean values for treatments are reported. Treatment means were separated using Tukey adjustment, and significant effects were declared at *p* < 0.05 with a tendency at *p* < 0.10.

## 3. Results

### 3.1. Thermal Pre-Treatment and Enzymatic Hydrolysis

There was no interaction between thermal pre-treatment and enzymatic hydrolysis for TRS, TPC, caffeine, and TEAC, as shown in Table 1.

No differences were observed for TPC, TEAC, and caffeine concentration among enzymes (Table 1). The mean losses of polyphenols, caffeine, and antioxidant compounds to the media were 5.6, 0.79, and 8.6 mg per gram of initial dry SCG, respectively.

Regarding TRS, a significant effect of the enzymatic hydrolysis was found, where Viscozyme^®^ released more TRS to the liquid fraction during hydrolysis than Celluclast^®^ (*p* = 0.013) or Ultraflo^®^ (*p* = 0.003), and Ultimase^®^ tended to release more TRS than Ultraflo^®^ (*p* = 0.076) (Table 1).

There was no significant interaction between thermal pre-treatment and enzymatic hydrolysis, nor a thermal pre-treatment effect on the chemical composition variables of the solid fractions of SCG (Table 2). Regarding the enzymatic hydrolysis treatments, no effect was observed for DM, CP, NDF, ADL, or NDICP in the SCG solid fraction (Table 2). However, enzymatic hydrolysis with Celluclast^®^, Ultimase^®^, and Viscozyme^®^ reduced the content of ash by 29% (*p* = 0.003), 29% (*p* = 0.003), and 33% (*p* < 0.001), respectively, compared with CTR, but no differences were observed for Ultraflo^®^ compared with CTR. All enzymes increased the content of ADICP (from 27 to 34%; *p* ˂0.001) compared with CTR without any significant differences among them. Viscozyme^®^ and Ultraflo^®^ also increased the content of ADF by 11% and 13%, respectively, compared with CTR (*p* < 0.05), whereas Celluclast^®^ and Ultimase^®^ showed only a tendency to increase it compared with CTR (*p* < 0.1).

Regarding SCG solid fraction in vitro digestibility values (Table 2), neither significant interaction between thermal pre-treatment and hydrolysis nor a thermal pre-treatment effect were observed. However, an effect of enzymatic hydrolysis on the IVOMD of SCG was observed (*p* = 0.001). In Table 2, it can be observed that all the enzymes reduced the IVOMD between 21% and 32% compared with the CTR, with no significant differences among enzymes.

Although enzymatic hydrolysis led to a decrease in IVOMD, SCFA concentration in the in vitro medium remained unaffected compared with CTR (*p* = 0.927; Table 3). There was no interaction between thermal pre-treatment and enzymatic hydrolysis for any of the measured fermentation products (Table 3). Enzymatic hydrolysis showed a tendency to increase acetic acid proportions; only Viscozyme^®^ significantly increased this parameter compared with CTR (62.5 vs. 60.8 mmol/100 mmol, *p* = 0.025), and all the tested enzymes decreased the propionic acid proportions (*p* < 0.001) by around 7 to 10% compared with CTR. All enzymes increased the acetic to propionic acid ratio (*p* ˂ 0.001) compared with CTR. Similarly, the thermal pre-treatment increased the acetic to propionic acid ratio (*p* ˂ 0.001) by 12% compared with CTR.

### 3.2. Mechanical Pre-Treatment and Enzymatic Hydrolysis with and without Removing the Liquid Phase

An interaction between the grinding pre-treatment and the hydrolysis process was observed to affect the concentrations of bioactive compounds in the SCG (Table 4).

Regarding TPC of the SCG samples, it was observed that the grinding process did not have a significant effect on TPC when the SCG samples were hydrolysed removing the liquid phase, whereas both fine grinding and coarse grinding increased TPC when SCG were not subjected to any hydrolysis, and fine grinding increased TPC when SCG were hydrolysed without removing the liquid phase (Table 4).

Regarding TEAC, similar results were observed in the SCG without hydrolysis where both fine and coarse grinding presented higher TEAC. When SCG were hydrolysed removing the liquid fraction, only fine grinding increased TEAC. When SCG were hydrolysed without removing the liquid fraction, no effect on TEAC was observed for fine grinding compared to unground, and coarse grinding even decreased it.

Caffeine content in SCG was significantly (*p* < 0.001) affected by the grinding pre-treatment and hydrolysis processes, although there was no significant interaction between them. The unground sample had a higher caffeine content than the fine (0.56 vs. 0.41%) and coarsely ground samples (0.56 vs. 0.42%). No differences were found between the fine and coarse grinding processes. Hydrolysed SCG samples with the liquid phase removed showed a lower caffeine content compared with non-hydrolysed ones (3.5 vs. 5.2 mg/g DM) and compared with SCG samples hydrolysed without removing the liquid phase (3.5 vs. 5.1 mg/g DM).

Regarding the TRS concentration of samples, an interaction between the grinding pre-treatment and the hydrolysis process was observed. When SCG samples were not hydrolysed, no differences between grinding processes were found. When samples were hydrolysed with and without removing the liquid phase, unground samples had lower TRS concentration than coarse or fine ground samples and coarse ground samples had lower TRS concentration than fine ground ones. The highest TRS was observed in the samples in which the liquid phase had not been removed (*p* < 0.001). 

A significant interaction between grinding and hydrolysis was observed for the ash content of the samples (Table 4). When the SCG were hydrolysed removing the liquid fraction, both grinding processes reduced the ash content compared with the unground samples, and fine grinding reduced it compared with coarse ground samples. However, when the samples were not hydrolysed both grinding types reduced ash to the same extent compared with unground samples, and when samples were hydrolysed without removing the liquid fraction only fine grinding reduced the ash content.

No interaction between grinding and hydrolysis processes was found for CP, NDICP, or ADICP content, but both the grinding and the hydrolysis process showed a significant effect. The unground SCG sample showed lower (150 g/kg DM) CP content compared with both fine (153 g/kg DM, *p* < 0.001) and coarse (152 g/kg DM, *p* < 0.001) grinding. However, the unground SCG sample (61 g/kg DM) showed greater CP content in the NDF fraction compared with fine (56 g/kg DM, *p* = 0.031) and coarse (54 g/kg DM, *p* = 0.003) grinding. A similar trend was observed for the CP content in the ADF fraction, where unground samples showed greater content compared with either fine (30 vs. 26 g/kg DM, *p* = 0.002) or coarse (30 vs. 27 g/kg DM, *p* = 0.046) grinding. The hydrolysis process with removing the liquid phase resulted in a reduction of the CP content compared with non-hydrolysed samples (150 vs. 153 g/kg DM, *p* < 0.001) and hydrolysed samples without removing the liquid phase (150 vs. 153 g/kg DM, *p* < 0.001), and no differences were observed between the two latter samples (*p* = 0.987). In addition, the hydrolysis process removing the liquid phase resulted in a greater NDICP fraction compared with hydrolysis without removing the liquid phase (33 vs. 27 g/kg DM, *p* = 0.004) and samples without hydrolysis (33 vs. 24 g/kg DM, *p* < 0.001), the difference being significant between the latter two (27 vs. 24 g/kg DM, *p* = 0.004). In addition, the hydrolysis process removing the liquid phase resulted in a greater NDICP fraction compared with hydrolysis without removing the liquid phase (66 vs. 55 g/kg DM, *p* < 0.001) and the samples without hydrolysis (66 vs. 55 g/kg DM, *p* < 0.001), the difference being non-significant between the latter two (*p* = 0.980).

Regarding the fibre fractions, only hydrolysis processes affected NDF content. Non-hydrolysed SCG samples showed greater NDF content compared with hydrolysis without removing the liquid phase (624 vs. 566 g/kg DM, *p* < 0.001), but lower content compared with hydrolysis removing the liquid phase (624 vs. 646 g/kg DM, *p* = 0.028). There were significant differences between hydrolysis removing the liquid phase and without removing the liquid phase (*p* < 0.001). Hydrolysis and grinding processes affected the ADF content in the SCG. Non-hydrolysed SCG samples showed lower ADF content compared with hydrolysis removing the liquid phase (375 vs. 454 g/kg DM, *p* < 0.001) and hydrolysis without removing the liquid phase (375 vs. 423 g/kg DM, *p* < 0.001). There were significant differences between hydrolysis removing the liquid phase and hydrolysis without removing the liquid phase (*p* < 0.001). The grinding process reduced ADF content after both fine (409 vs. 440 g/kg DM, *p* < 0.001) and coarse (402 vs. 440 g/kg DM, *p* < 0.001) grinding compared with unground samples, and the differences between fine and coarse grinding were not significant (*p* = 0.623).

A significant interaction was observed for ADL content only when the samples were hydrolysed removing the liquid phase; lower ADL contents were found when the sample was coarse ground compared with fine (*p* < 0.001) or unground samples (*p* = 0.018). 

Regarding IVOMD, Table 5 reveals that no significant interaction between grinding pre-treatment and hydrolysis was observed. However, both grinding pre-treatment and hydrolysis affected the IVOMD values of the SCG.

Table 5 shows that hydrolysis of SCG removing the liquid fraction reduced IVOMD compared either with unprocessed SCG (272 vs. 350 g/kg OM, *p* < 0.001) or hydrolysed SCG without removing the liquid fraction (272 vs. 364 g/kg OM, *p* < 0.001). However, no differences were found between non-hydrolysed samples and hydrolysed samples without removal of the liquid phase (*p* = 0.403). Fine grinding (429 g/kg OM, *p* < 0.001) and coarse grinding (297 g/kg OM, *p* = 0.003) processes both resulted in increased IVOMD compared with the unground sample (261 g/kg OM). In addition, fine grinding improved IVOMD compared with coarse grinding (*p* < 0.001).

Similar results were observed for fermentation parameters, where no significant interaction between grinding and hydrolysis was observed for SCFA production. Fine grinding increased SCFA production compared with either coarse grinding (4.52 vs. 3.85 mmol/100 mL, *p* < 0.001) or unground samples (4.52 vs. 3.73 mmol/100 mL, *p* < 0.001).

A significant interaction between grinding and hydrolysis was found for the main SCFA produced during fermentation of the samples.

Regarding acetic acid, no significant differences between grinding conditions were found when the samples were not subjected to hydrolysis. However, when hydrolysis removing the liquid phase process was applied, both fine and coarse grindings reduced the acetic proportions compared with the unground sample. When the hydrolysis without removing the liquid phase process was applied, only fine grinding reduced the acetic proportions compared with the unground sample. Propionic molar proportions increased in the fine ground samples regardless of the hydrolysis process. Butyric molar proportions were not affected by the grinding process when hydrolysis processes were applied either with or without removing the liquid phase. However, when not subjected to hydrolysis, butyrate molar proportions were reduced after samples were subjected to fine grinding, compared with either coarse or unground samples. Fine grinding resulted in a lower acetic:propionic acid ratio compared with either coarse grinding (2.12 vs. 2.48, *p* < 0.001) or unground samples (2.12 vs. 2.55, *p* < 0.001), with differences between the latter not being significant (*p* = 0.106). Subjecting SCG samples to hydrolysis without removing the liquid phase resulted in a reduced acetic:propionic acid ratio compared with hydrolysis removing the liquid phase (2.32 vs. 2.42, *p* = 0.002) or samples without hydrolysis (2.32 vs. 2.42, *p* = 0.007), and the differences between the latter were not significant (*p* = 0.929).

## 4. Discussion

Spent coffee grounds have been included in ruminant rations at doses up to 10% of the concentrate (3% of total diet) for dairy sheep [11] and up to 5% of the concentrate (2% of total diet) for dairy cows [2] without impairing productive performance. In addition, De Otalora et al. [11] observed some improvement on productive performance with the SCG diet, which could be related to the effect of the secondary compounds present in the SCG on the rumen microbial populations [12].

Although these results are promising, the aim of a “circular economy” approach should be to maximize the inclusion of this by-product in ruminants’ rations. However, other studies have reported that the low fibre digestibility of SCG impaired animal performance when this by-product was included in the ration in a much higher dose [30,31,32].

To overcome these constraints, different valorisation strategies were tested in the present study with the aim of attacking the lignocellulosic bonds and fibre fraction of the SCG and thereby improving their digestibility.

In the first experiment, contrary to other studies in the literature [33,34], thermal pre-treatment did not improve SCG nutritive value. Autoclaving of the SCG in the conditions described in this experiment (121 °C, 15 min) appeared to be ineffective for breaking the lignocellulosic bounds in the SCG. These results could be due to a low rate of cellulose hydrolysis and, therefore, low TRS release at the autoclaving temperature used. Other authors working with lignocellulosic biomass have reported that reaction temperature when applying thermal pre-treatments significantly affects the characteristics of the solid product obtained [35]. Therefore, it is possible either that the temperature applied in the present study was not high enough or the time was not long enough to obtain an effect from the thermal pre-treatment.

In contrast, enzymatic hydrolysis affected the nutritive value and digestibility of SCG. The hydrolysis processes carried out involved addition of water that was subsequently removed when the hydrolysis was finished. It was noteworthy that according to the data measured in the liquid fraction, the enzymatic hydrolysis process did not seem to compromise the concentration of bioactive compounds in solid SCG intended for animal nutrition. This issue is of interest, as mentioned previously, due to the potential beneficial effects of bioactive compounds present in SCG on animals’ productive performance and health [11].

Release of TRS to the liquid medium was observed to differ between the various enzymes used for the hydrolysis processes. These differences between enzymes in the release of TRS are probably related to the higher enzymatic activity of Viscozyme ^®^ and Ultraflo ^®^.

However, as the liquid fraction is removed, it is expected that this release of TRS to the liquid fraction during the hydrolysis processes would lead to a loss of TRS in the solid fraction intended for animal nutrition compared with the unprocessed SCG (CTR). In the first experiment, TRS in the solid fraction were not measured, but this hypothesis is corroborated by the changes observed in the proportions of other components of the solid fraction. Indeed, a concentration of insoluble and more recalcitrant compounds (ADF and ADICP) in the solid part was observed, which could be partially explained by the release of soluble compounds in the liquid phase followed by its subsequent elimination. Such an effect is not surprising, since studies dealing with the improvement in TRS release to the liquid phase in biomass-to-bioethanol processes using lignocellulosic materials have shown the efficiency of enzymatic hydrolysis in releasing TRS to the liquid media [14,36,37,38,39]. Moreover, a lower concentration of ash was observed after the hydrolysis, which could be due to the draining of minerals to the liquid phase during the hydrolysis process and their loss when this fraction was removed.

Digestibility of feedstuffs is an important issue to consider when formulating a diet, and is known to be closely related to their physicochemical composition. In this sense, the results obtained for the physicochemical composition of the solid fraction of the SCG and the TRS concentration of the liquid phase after the hydrolysis processes are in line with those observed for the digestibility of the solid fraction.

Fibre concentration is one of the main important factors affecting dry matter digestibility, especially in relation to its level of lignification [40,41,42]. Reducing concentration of sugars in a feedstuff is also of interest, since these are rapidly fermented in the rumen, yielding microbial cells, organic acids, gas, and microbial glycogen. For example, glucose and fructose were completely fermented within 4–6 h in the rumen [43] and TRS concentration in a feedstuff is therefore related to its digestibility.

Although reduced digestibility was observed with the enzymatic hydrolysis, production of total SCFA during ruminal fermentation was not affected. This is surprising, since SCFA production during fermentation is positively associated with the amount of organic matter fermented by ruminal microorganisms [44]. However, Hvelplund [45] observed that in situations where microbial synthesis efficiency was low, the fermentation products increased in relation to the amount of substrate digested. Therefore, it could be said that when the SCG in the present study were hydrolysed, the microbial growth in the in vitro systems may have been limited, and this issue also affected the digestibility values.

The physicochemical characteristics of a feedstuff also influence the rumen fermentative process and, therefore, the SCFA produced as a consequence. In general, raw material with an elevated ADF value leads to a fermentation process in the rumen with acetic acid as a main final product in detriment of propionic production, which corroborates the results observed in this experiment.

The effects observed for the enzymatic hydrolysis are relevant in the context of ruminants’ nutrition, since a decrease in digestibility and a shift towards less efficient fermentation routes with proportionally more acetic and less propionic acid is not desirable.

Therefore, taking into account the results of this first experiment, a different pre-treatment method (grinding) was tested in a second experiment to try to break the lignocellulosic bounds present in the SCG fibre fraction. Furthermore, considering the influence of the release of soluble compounds to the liquid fraction on the physicochemical characteristics and digestibility of the solid fraction, an enzymatic hydrolysis process that did not include removal of the liquid fraction was also tested.

In order to study the effect of the enzymes, eliminating the dilution effect caused by solid–liquid separation, Ultimase^®^ and Viscozyme^®^ were selected for the hydrolysis because they showed higher release of TRS to the liquid fraction associated with higher enzymatic activity.

In this second experiment, grinding pre-treatment and EH showed an effect on SCG physicochemical characteristics and digestibility. Moreover, an interaction between them was observed for some of the measured variables.

Regarding the proportion of secondary compounds in the SCG, contrary to the results observed in the first experiment, effects of the grinding pre-treatment and hydrolysis process on the concentrations of bioactive compounds in the SGC were observed in the second experiment.

The effect of grinding on the caffeine content in the brew and, therefore, in the SCG has previously been analysed in the literature [46], with conclusions in line with our results that the finer the grinding the more caffeine appears in the brew and the less in the remaining SCG. Concerning EH, the results suggested that some of the caffeine may have been released to the liquid fraction during the hydrolysis process and was removed in the hydrolysed samples when the liquid phase was removed, and that this effect disappeared when the liquid phase was not removed after the hydrolysis.

The concentrations of TPC and TEAC indicated an interaction between the grinding pre-treatment and EH. The mechanical pre-treatment increased their concentrations in the SCG, but this effect disappeared when the liquid fraction was removed after the hydrolysis process. This confirmed the hypothesis of the first experiment concerning loss of compounds by solubilisation in the liquid during EH.

Coffee polyphenols have been variously studied for their antioxidant properties [47]. Polyphenols in plant materials are closely connected to the plant cell wall structure [48] and any attempt to break the lignocellulosic bounds of the cell wall may cause a release of these bioactive compounds. In this sense, grinding pre-treatment seemed to achieve this objective thereby improving the antioxidant capacity. However, TEAC results observed for hydrolysis with and without removing the liquid phase were not so clearly related to the results obtained for TPC.

There is no clear explanation for these data observed in the hydrolysis processes. Antioxidant compounds have different mechanisms of action correlated with structural specificity [49]. Therefore, depending on the type of compounds present in the sample, the results may differ due to the method of analysis [50]. In this sense, several studies have shown that there is not always a linear relationship between antioxidant capacity and TPC determined by the Folin–Ciocalteu method [51]. Another possible explanation is the presence in the SCG of non-phenolic compounds with TEAC that were not analysed in the current study, such as melanoidins. Melanoidins are compounds with recognized antioxidant capacity that are formed due to Maillard reactions during the processing of coffee [52]. These melanoidins are not fully extractable nor digestible, which may interact with their release to the liquid fraction during hydrolysis processes [53].

Regarding the nutritive value of SCG, the interaction observed between mechanical pre-treatment and EH for TRS concentration indicates that grinding pre-treatment before the hydrolysis process was effective in increasing the surface area of the SCG and facilitating contact with enzymes [13], therefore increasing the TRS content in the SCG samples. Some of these TRS were released to the liquid fraction, and thus the SCG sample hydrolysed without removing the liquid fraction showed the highest content of TRS per g of DM. Moreover, this also indicates that EH has the potential to increase digestibility by degrading fibre fractions, as reported in the literature [19].

The results concerning SCG ash content agreed with those obtained in the first study and with the hypothesis that some minerals were solubilized in the liquid fraction during the hydrolysis processes and removed from the solid fraction. These results also indicate that as the grinding became more intense the release of minerals increased, especially in the hydrolysed samples.

Mechanical pre-treatment showed other results of interest for animal nutrition. The grinding pre-treatment could increase the CP of SCG intended for animal feeding and reduce the amount of CP that it is attached to the fibrous fraction and is more difficult for the animal to digest.

However, EH exerted the opposite results. The slight decrease in CP and increase in protein linked to fibre observed with the hydrolysis process is not positive from an animal nutrition point of view, as previously mentioned, but these negative effects disappeared when the liquid fraction was retained after the hydrolysis.

Regarding the fibre fractions, EH affected NDF content. No such effect was observed in the first experiment, when NDF or CP were not affected by the hydrolysis. It may be that the two selected enzymes used together could digest the SCG samples to a greater extent, leading to less fibre-associated protein and releasing more simple carbohydrates to the media and concentrating fibre fractions in the solid phase. It is possible that this effect was counterbalanced when the samples were reconstituted with the liquid fraction.

Results obtained for the physicochemical composition of the solid fraction of the SCG after the grinding pre-treatments and the hydrolysis processes are in line with the IVOMD results observed. Moreover, as stated previously, the physicochemical characteristic of a feedstuff also influences the rumen fermentative process and, therefore, the SCFA produced as a consequence. In this context, the effects on the composition of the fibre fraction observed in this experiment could have led to the results obtained for individual SCFA proportions during in vitro fermentation.

Results obtained in the second experiment showed that the grinding pre-treatment contributed to the breakdown of the lignocellulosic bounds present in the SCG fibre fraction, and that this effect depended on the particle size. Previous studies have proven the efficacy of grinding as a pre-treatment for lignocellulosic materials [54]. From the animal nutrition point of view, the decrease in more recalcitrant fibre components and its influence on improving the digestibility of SCG and the fermentation products obtained suggest that such pre-treatment may be a very interesting option for valorisation of this by-product. Furthermore, the results observed for the hydrolysis processes indicate that the release of TRS and other substances of interest to the liquid phase during the hydrolysis led to a SCG solid fraction intended for animal nutrition that had poorer physicochemical characteristics and lower digestibility, as was observed in the first experiment. This is not desirable, and it opposes the main objective of this work. Conversely, when the liquid fraction of the hydrolysis process was not removed, these negative effects were avoided. In this case, the physicochemical composition of the obtained SCG was more interesting in terms of animal nutrition than the SCG without processing. However, these changes in the physicochemical composition did not lead to greater IVOMD as had been expected, with IVOMD values similar to those of the unprocessed SCG.

These results could be explained by the inherent enzymatic activity of the rumen microorganisms. Ruminant animals harbour a diverse and complex microbial ecosystem capable of digesting and fermenting feedstuffs rich in fibre. These rumen microbes have developed the ability to efficiently use complex plant polymers such as, for example, cellulose and hemicellulose. It is known that degradation and fermentation of structural carbohydrates is accomplished by a cascade of activities carried out by the diverse microbial enzymes that exist in the rumen (cellulases, xylanases, β-glucanases, pectinases, amylases, proteases, phytases, tannases, etc.) [55]. Therefore, in light of these results, the hydrolysis of fibre components under these experimental conditions prior to ruminal fermentation did not improve ruminal digestibility, because rumen microorganisms could counterbalance the benefits of this hydrolysis with their enzymatic activity to attain similar digestibility values. However, this enzymatic process could be of interest for monogastric animals, which lack this type of effective microbial digestion in their guts. Thus, this could be a topic of interest for future works.

Although an interaction between grinding pre-treatment and the hydrolysis process was found for many of the measured variables, the limited results observed with the hydrolysis processes minimize the practical opportunities for combined application of these treatments. However, grinding alone could be an interesting strategy for SCG valorisation. Maximizing the efficiency of plant cell wall material degradation in the rumen has become an important goal in modern livestock production [55]. It is known that the insolubility, structural complexity, and initial inaccessibility of cell wall components often limit the extent to which they are fermented in the rumen [56], and the grinding pre-treatment seemed to succeed in improving this accessibility.

## 5. Conclusions

This study determined the effect of thermal and mechanical pre-treatments combined with enzymatic hydrolysis to improve spent coffee grounds’ nutritional value as an ingredient for ruminants’ diets.

The hydrolysis process with different cellulolytic enzymes involved a release of valuable compounds, such as sugars, polyphenols, and other elements with antioxidant activity into the liquid fraction, resulting in a less valuable raw material. In addition, the action of the enzymes on the solid fraction was counteracted by the action of the ruminal bacteria. Thermal pre-treatment of the SCG appeared to be ineffective in improving the breakdown of lignocellulosic bonds and thus improving digestibility. In contrast, grinding pre-treatment improved the coffee grounds’ digestibility and the fermentative process in the rumen.

In conclusion, extra grinding is presented as the most powerful technological choice to improve the digestibility of spent coffee grounds for their reintroduction into the value chain.

## Figures and Tables

**Table 1:** Effects of enzymatic hydrolysis on the concentrations of total reducing sugars, polyphenols expressed as gallic acid equivalent (GAE), caffeine, and antioxidant capacity expressed as trolox equivalent antioxidant capacity (TEAC) in the liquid phase of the SCG hydrolysates with and without thermal pre-treatment. Results are expressed as mg of compound per g of initial dry SCG (n = 3).

Item (mg/g DM)	Treatment								SEM		*p*-Value	
	With				Without					Tre	Enz	Tre*Enz
	1	2	3	4	1	2	3	4				
Reducing sugars	8.0	9.2	11.2	6.2	7.1	9.2	12.9	6.8	2.07	0.905	0.003	0.697
Polyphenols	5.5	5.6	5.8	6.4	5.4	5.4	5.3	5.8	0.93	0.397	0.549	0.956
Caffeine	0.76	0.85	0.68	0.64	0.75	0.96	0.79	0.87	0.140	0.072	0.164	0.541
Antioxidant Capacity	8.5	9.4	8.3	8.6	9.0	8.3	8.6	8.3	1.17	0.784	0.910	0.644

With: SCG with thermal pre-treatment; Without: SCG without thermal pre-treatment; 1: Celluclast^®^; 2: Ultimase^®^; 3: Viscozyme^®^; 4: Ultraflo^®^; SEM: standard error of the mean; Tre: pre-treatment; Enz: enzyme; GAE: gallic acid equivalent; TEAC: trolox equivalent antioxidant capacity; SCG: spent coffee grounds; DM: dry matter.

**Table 2:** Effects of enzymatic hydrolysis on the chemical composition and in vitro organic matter digestibility of the solid part of the SCG with and without thermal pre-treatment.

Item (g/kg DM)	Treatment										SEM	*p*-Value		
	With					Without						Tre	Enz	Tre*Enz
	1	2	3	4	CTR	1	2	3	4	CTR				
DM	844	884	854	860	924	879	915	886	873	917	45.8	0.310	0.386	0.975
Ash	12.0	11.4	10.8	14.7	14.8	10.7	11.2	10.4	12.0	16.9	1.24	0.386	˂0.001	0.200
CP	148	152	154	148	153	150	150	152	150	154	2.9	0.820	0.092	0.632
NDF	696	692	684	686	689	688	661	681	687	680	21.8	0.311	0.804	0.739
ADF	424	416	424	446	396	418	423	434	428	378	10.1	0.465	0.013	0.488
ADL	178	186	182	171	164	170	172	172	175	165	14.4	0.389	0.763	0.854
NDICP	69	66	66	67	57	63	60	61	72	56	5.7	0.355	0.102	0.384
ADICP	33	31	32	33	26	33	31	33	32	23	1.6	0.259	˂0.001	0.614
IVOMD(g/kg OM)	219	198	203	209	287	188	217	218	213	276	18.1	0.959	0.001	0.169

With: SCG with thermal pre-treatment; Without: SCG without thermal pre-treatment; 1: Celluclast^®^; 2: Ultimase^®^; 3: Viscozyme^®^; 4: Ultraflo^®^; CTR: control (unprocessed SCG); SEM: standard error of the mean; Tre: pre-treatment; Enz: enzyme; DM: dry matter; CP: crude protein; CF: crude fat; NDF: neutral detergent fibre; ADF: acid detergent fibre; ADL: acid detergent lignin; NDICP: crude protein in NDF; ADICP: crude protein in ADF; IVOMD: in vitro organic matter digestibility; OM: organic matter.

**Table 3:** Effect of enzymatic hydrolysis on main short chain fatty acids produced during ruminal fermentation of SCG with and without thermal pre-treatment.

Item	Treatment										SEM	*p*-Value		
	With					Without						Tre	Enz	Tre*Enz
	1	2	3	4	CTR	1	2	3	4	CTR				
SCFA (mmol/L)	41.5	40.8	40.7	40.9	43.6	41.6	44.3	43.0	42.4	42.5	2.6	0.284	0.927	0.740
Individual SCFA proportions (mmol/100 mmol)														
Acetic	62.3	62.2	62.7	62.4	60.9	61.6	61.8	62.3	61.9	60.6	0.63	0.099	0.051	0.989
Propionic	18.8	19.1	18.6	18.8	20.7	19.0	19.5	19.0	19.3	20.9	0.37	0.062	˂0.001	0.996
Butyric	11.7	11.4	11.5	11.4	11.2	11.5	11.4	11.5	11.7	11.1	0.41	0.988	0.758	0.929
Acetic:propionic	3.43	3.37	3.47	3.43	3.12	3.35	3.30	3.40	3.32	3.02	0.041	˂0.001	˂0.001	0.938

With: SCG with pre-treatment; Without: SCG without pre-treatment; 1: Celluclast®; 2: Ultimase®; 3: Viscozyme®; 4: Ultraflo®; CTR: control (unprocessed SCG); SEM: standard error of the mean; Tre: treatment; Enz: enzyme; SCFA: short chain fatty acid; FA: fatty acids.

**Table 4:** Effect of enzymatic hydrolysis and grinding on the nutritional value of spent coffee grounds.

Item (g/kg DM)	Treatment									SED	*p*-Value		
	Hyd			HydC			Unp				H	G	H*G
	FG	CG	UnG	FG	CG	UnG	FG	CG	UnG				
DM	970	979	977	977	976	968	960	977	958	15.3	0.347	0.382	0.743
Ash	1.4 a	2.0 b	2.6 c	1.7 a	2.1 ab	2.2 b	1.3 a	1.7 a	2.7 b	0.158	0.123	<0.001	<0.001
CP	151	151	148	154	153	151	154	153	150	11.4	<0.001	<0.001	0.582
ADF	461	430	470	409	416	443	357	362	406	14.8	<0.001	<0.001	0.112
ADL	195 a	171 b	188 a	175	170	184	156	149	159	5.3	<0.001	<0.001	0.037
NDF	652	640	645	538	583	578	630	628	616	15.8	<0.001	0.422	0.190
ADICP	32	31	35	24	27	30	22	24	25	1.9	<0.001	0.003	0.213
NDICP	66	60	68	50	53	59	50	48	56	3.9	<0.001	0.003	0.371
Polyphenols (mg/g DM)	10.8	9.6	10.0	18.1 b	13.1 a	14.5 a	16.3 b	16.7 b	13.3 a	00.70	<0.001	<0.001	<0.001
Antioxidant activity (mg TEAC/g DM)	13.4 b	10.5 a	11.1 a	15.9 b	13.7 a	16.6 b	15.1 b	14.3 b	12.0 a	0.66	<0.001	<0.001	<0.001
Caffeine (mg/gDM)	3.02	3.19	4.33	4.63	4.58	6.15	4.76	4.75	6.18	0.107	<0.001	<0.001	0.058
Sugars (mg/g DM)	35 c	30 b	20 a	84 c	54 b	35 a	10.6	9.7	9.6	1.56	<0.001	<0.001	<0.001

Hyd: hydrolysed spent coffee grounds with removal of the liquid fraction; HydC: hydrolysed spent coffee grounds without removing the liquid fraction; Unp: unprocessed spent coffee grounds; UnG: non-ground spent coffee grounds; FG (ground to 100 µm); CG (ground to 250 µm); SED: standard error of the difference; H: effect of the hydrolysis type; G: effect of the grinding pre-treatment; DM: dry matter; OM: organic matter; EE: ether extract; CP: crude protein; NDF: neutral detergent fibre; ADF: detergent acid fibre; ADL: detergent acid lignin; NDICP: crude protein in FND; ADICP: crude protein in ADF. Means within a row for grinding type and hydrolysis indicated by different letters differ significantly (*p* < 0.05).

**Table 5:** Effect of enzymatic hydrolysis and grinding on in vitro digestibility and on fermentation parameters of spent coffee grounds.

Item	Treatment									SED	*p*-Value		
	Hyd			HydC			Unp				**H**	**G**	H*G
	FG	CG	UnG	FG	CG	UnG	FG	CG	UnG				
IVOMD (g/kg OM)	351	248	216	479	323	289	456	318	276	18.0	<0.001	<0.001	0.192
SCFA (mmol/L)	4.11	3.78	3.46	4.49	3.74	3.62	4.97	4.02	4.10	0.157	<0.001	<0.001	0.071
Individual SCFA proportions (mmol/100 mmol)													
Acetic	57.5 b	57.7 b	59.9 a	57.2 b	60.1 a	60.2 a	57.6 a	59.3 a	58.9 a	0.734	0.173	<0.001	0.033
Propionic	26.1 a	24.2 b	23.2 b	28.4 a	24.5 b	24.3 b	27.2 a	23.4 b	23.2 b	0.382	<0.001	<0.001	0.001
Butyric	10.5 a	11.3 a	10.6 a	9.40 a	9.91 a	9.91 a	9.98 b	11.1 a	11.3 a	0.298	<0.001	<0.001	0.033
Acetic: propionic	2.21	2.43	2.59	2.02	2.45	2.49	2.13	2.56	2.58	0.059	0.004	<0.001	0.082

Hyd: hydrolysed spent coffee grounds with removal of the liquid fraction; HydC: hydrolysed spent coffee grounds without removing the liquid fraction; Unp: unprocessed spent coffee grounds; UnG: non ground spent coffee ground; FG (ground to 100 µm); CG (ground to 250 µm); SED: standard error of the difference; H: effect of the hydrolysis type; G: effect of the grinding pre-treatment; IVOMD: in vitro organic matter digestibility; OM: organic matter; SCFA: short chain fatty acid. Means within a row for grinding type and hydrolysis indicated by different letters differ significantly *p* < 0.05).

## Data Availability

The datasets used and analysed during the current study are available from the corresponding author on reasonable request.

## Glossary

ADF	Acid Detergent Fibre
ADICP	Acid Detergent Insoluble Crude Protein
ADL	Acid Detergent Lignin
CP	Crude Protein
CTR	Control
DM	Dry Matter
DPPH	2,2-Diphenyl-1-picrylhydrazyl
EH	Enzymatic Hydrolysis
FEFAC	European Feed Manufacturers’ Federation
GAE	Gallic Acid Equivalent
HORECA	Hotels, Restaurants and Catering industry
ICO	International Coffee Organization
IVOMD	In Vitro Organic Matter Digestibility
NDF	Neutral Detergent Fibre
NDICP	Neutral Detergent Insoluble Crude Protein
OM	Organic Matter
SCFA	Short Chain Fatty Acids
SCG	Spent Coffee Ground
SED	Standard Error of the Difference
SEM	Standard Error of the Mean
TEAC	Trolox Equivalent Antioxidant Capacity
TPC	Total Polyphenol Content
TRS	Total Reducing Sugars
